# Invadolysin acts genetically via the SAGA complex to modulate chromosome structure

**DOI:** 10.1093/nar/gkv211

**Published:** 2015-03-16

**Authors:** Shubha Gururaja Rao, Michal M. Janiszewski, Edward Duca, Bryce Nelson, Kanishk Abhinav, Ioanna Panagakou, Sharron Vass, Margarete M.S. Heck

**Affiliations:** University of Edinburgh, Queen's Medical Research Institute, University/BHF Centre for Cardiovascular Science, 47 Little France Crescent, Edinburgh EH16 4TJ, UK

## Abstract

Identification of components essential to chromosome structure and behaviour remains a vibrant area of study. We have previously shown that invadolysin is essential in *Drosophila*, with roles in cell division and cell migration. Mitotic chromosomes are hypercondensed in length, but display an aberrant fuzzy appearance. We additionally demonstrated that in human cells, invadolysin is localized on the surface of lipid droplets, organelles that store not only triglycerides and sterols but also free histones H2A, H2Av and H2B. Is there a link between the storage of histones in lipid droplets and the aberrantly structured chromosomes of *invadolysin* mutants? We have identified a genetic interaction between *invadolysin* and *nonstop*, the de-ubiquitinating protease component of the SAGA (Spt-Ada-Gcn5-acetyltransferase) chromatin-remodelling complex. *invadolysin* and *nonstop* mutants exhibit phenotypic similarities in terms of chromosome structure in both diploid and polyploid cells. Furthermore, *IX-14^1^*/*not^1^* transheterozygous animals accumulate mono-ubiquitinated histone H2B (ubH2B) and histone H3 tri-methylated at lysine 4 (H3K4me3). Whole mount immunostaining of *IX-14^1^*/*not^1^* transheterozygous salivary glands revealed that ubH2B accumulates surprisingly in the cytoplasm, rather than the nucleus. Over-expression of the Bre1 ubiquitin ligase phenocopies the effects of mutating either the *invadolysin* or *nonstop* genes. Intriguingly, *nonstop* and mutants of other SAGA subunits (*gcn5, ada2b* and *sgf11*) all suppress an invadolysin-induced rough eye phenotype. We conclude that the abnormal chromosome phenotype of *invadolysin* mutants is likely the result of disrupting the histone modification cycle, as accumulation of ubH2B and H3K4me3 is observed. We further suggest that the mislocalization of ubH2B to the cytoplasm has additional consequences on downstream components essential for chromosome behaviour. We therefore propose that invadolysin plays a crucial role in chromosome organization via its interaction with the SAGA complex.

## INTRODUCTION

The visible changes that chromosomes undergo through the cell cycle have been the subject of intense cytological analysis for over a century. Recently, many powerful techniques have been brought to bear on the subject of the reversible cycle of chromosome condensation and decondensation. Biochemical, physical, genetic and cell biological approaches have all served to identify a myriad of structural and regulatory components whose detailed roles are still being elucidated (1).

Chromatin condensation is particularly evident during mitosis and apoptosis, whereas chromatin relaxation is associated with transcription, replication, repair and recombination (2). Post-translational modifications of histones, e.g. acetylation, methylation, phosphorylation and ubiquitination, play key roles in chromatin dynamics (3,4). Acetylation of histones is generally associated with ‘relaxing’ or opening chromatin in order to support the access of transcriptional machinery to genomic loci (5). In contrast, histone methylation and ubiquitination have been linked to both gene activation and repression (6). Specific phosphorylation signatures have been identified for both mitosis and apoptosis. The enzymes that catalyse histone modification are often organized into large multi-subunit complexes, which facilitate enzymatic activity and enhance substrate specificity.

We previously reported the identification of invadolysin, predicted to be a metalloprotease, which links the processes of mitosis and cell migration (7). *invadolysin* mutant larvae display defects in mitotic chromosome packaging, appearing hypercondensed in length but with a ‘fuzzy’ or ragged appearance at the periphery. The chromosome phenotype was distinct from other chromosome condensation mutations we analysed (8–10), and *invadolysin* mutant neuroblasts also exhibited defects in spindle assembly and nuclear envelope dynamics. The active migration of primordial germ cells was also affected in *invadolysin* mutant fly embryos, this being consistent with the localization of invadolysin at the leading edge of migrating human macrophages (7). The human invadolysin protein is localized to intracellular ring-like structures, which we subsequently identified as lipid droplets (11). A functional role for invadolysin in lipid metabolism is suggested by a decreased triglyceride to protein ratio in mutant larvae (11). While the gene is conserved from bacteria to higher eukaryotes, it is curiously absent from archaea, fungi, and viruses. There are four splice variants of human invadolysin, though so far only one has been described in *Drosophila* (11).

Proteases are crucial for homeostasis through the regulation of inter-protein and inter-cellular interactions. Metalloproteases are a class of proteases that require either zinc or calcium for their activity (12). Metalloproteases from the MA clan contain a short consensus HEXXH sequence—the two histidines act as ligands for the zinc ion, while glutamate acts as a catalytic base (13). The M8 metzincin family (to which invadolysin belongs) also encodes a more C-terminal histidine that is a third ligand for zinc. Metalloproteases are involved in crucial processes affecting cell adhesion, cell migration, tissue remodelling and differentiation (14). Metalloproteases have been implicated in numerous regulatory roles emphasizing their diverse functions and biological importance.

In an attempt to better understand invadolysin, we describe herein a second site non-complementation screen performed in *Drosophila* to identify genes interacting with invadolysin. We identified the gene responsible for non-complementation within a synthetic lethal deficiency as the ubiquitin protease *nonstop* (a component of the multi-subunit SAGA chromatin-modifying complex (15)). Importantly, *invadolysin* and *nonstop* mutants showed strikingly similar chromosome defects at both the cytological and molecular levels in diploid and polyploid cells. Our results suggest that the abnormal chromosome phenotype might be attributed to increased levels of ubiquitinated histone H2B. Thus, we have discovered a role for invadolysin, via the activity of the SAGA complex, in the regulation of chromosome structure.

## MATERIALS AND METHODS

### Maintenance of *Drosophila* stocks

All fly stocks were maintained at 25°C on standard medium unless otherwise stated. The Canton S strain served as the wild type stock, and is indicated as ‘wt’ through the manuscript. The deficiency collection was from DrosDel (Szeged) and Gal4 driver flies were from the Bloomington Stock Centre. *Nonstop* mutant flies were a kind gift from I. Salecker and B. Poeck. UAS-*Bre1* flies were a generous gift from M. Bienz and S. Bray. Genetic crosses were performed using virgin female flies collected as soon as possible after eclosion.

### Second site non-complementation screen

The DrosDel deficiency kit was used as these deficiencies have molecularly defined breakpoints and are originally derived from an isogenic stock. Five to ten virgin females from *IX-14^1^* mutant stocks over TM3 or TM6B were crossed with two to three males from each of the 225 DrosDel deficiency lines. Flies were left to mate and lay eggs for 5 days at room temperature. The parents were removed from the vials and the progeny were allowed to emerge in the same vial. These crosses were carried out at room temperature and after 12 days, the eclosed flies were scored for the presence or absence of balancer chromosomes. Males and females were counted separately in order to investigate if there were any gender specific phenotypes.

### Preparation of larval samples for SDS-PAGE

Five to thirty wandering third instar larvae of the appropriate genotype were removed from culture bottles and transferred to 1.5 ml Eppendorf tubes and rinsed 3× in EBR (Ephrussi-Beadle Ringer's solution: 130 mM NaCl, 4.7 mM KCl, 1.9 mM CaCl_2_, 10 mM HEPES, pH 6.9). One hundred fifty microlitres of cold lysis buffer (EBR, 10 mM ethylenediaminetetraacetic acid (EDTA), 10 mM dithiothreitol (DTT), 10 μg/ml each of chymostatin, leupeptin, antipain and pepstatin [CLAP], 1 mM phenylmethanesulfonyl fluoride [PMSF], and 1 unit aprotinin) was added to the tubes. The larvae were then homogenized using a hand pestle starting at lowest speed and gently increasing the speed to the maximum for ∼2 min on ice. An additional 150 μl of cold lysis buffer was added and samples were sonicated (3× for 5 s at 5–10 amplitude microns power). One hundred fifty microlitres of hot (70°C) 3× sodium dodecyl sulphate-polyacrylamide gel electrophoresis (SDS-PAGE) sample buffer +10 μl of 1M DTT was added to the homogenate and the tube was placed at 100°C for 10 min. Particulate matter was centrifuged at 13 000 rpm for 2 min at 4°C in a benchtop centrifuge, and the supernatant transferred to a fresh tube. Samples were stored at −20°C until required. Protein samples were resolved by SDS-PAGE on bis–tris pre-cast gels (Invitrogen) at 125 V for ∼90 min, transferred to nitrocellulose membranes (Schleicher & Schuell), and processed for immunoblotting.

### Immunoblotting

Non-specific binding to nitrocellulose membranes was blocked in TBS-Tw (Tris-buffered saline with 0.05% Tw-100) and 5% (w/v) semi-skim powdered milk (Sainsbury's) for 30 min at room temperature (RT). Membranes were then washed 3× for 5 min with TBS-Tw. The appropriate dilution of primary antibodies was prepared in TBS-Tw and 5% (w/v) BSA and membranes were blotted with primary antibodies overnight at 4°C. Following overnight incubation membranes were washed three times for 5 min with TBS-Tw, the membranes were then incubated in the appropriate HRP-linked secondary antibody for 1 h in TBS-Tw and 5% (w/v) semi-skim powdered milk at RT. Finally, the membranes were washed 6× for 5 min with TBS-Tx followed by two rinses in TBS. Immune-complexes were detected by enhanced chemiluminescence (ECL, Amersham Biosciences).

Primary antibodies and dilutions used in immunoblotting experiments were as follows: ubH2B @ 1:250 (Medimabs), H3K4me3 @ 1:1000 (Cell Signalling), H3K4me2 @ 1:1000 (Cell Signalling), H3K4me1 @ 1:1000 (Cell Signalling), H3K9/14ac @ 1:1000 (Cell Signalling), H3K18ac @ 1:500 (Cell Signalling), histone H3 @ 1:1000 (Abcam), histone H2B @ 1:1000 (Abcam), α-tubulin @ 1:2000 (Sigma).

### DAPI staining of larval brains

Brains were dissected from second or third instar larvae in EBR as described above and fixed twice in 45% acetic acid, for 90 s each, in a dissecting dish. The brains were then transferred to a drop of 45% acetic acid (5 μl per drop) on a siliconized coverslip and a poly-l-lysine slide was inverted on top of the coverslip (total fixation should not exceed 3 min). The brains were squashed and flash-frozen by dipping in liquid nitrogen. The coverslip was breathed on to warm and flicked off with a razor blade and immersed immediately in a Coplin jar containing phosphate buffered saline (PBS) for 5 min to remove the acetic acid. The slide was transferred to a jar containing 0.1 μg/ml 4′,6-diamidino-2-phenylindole (DAPI) in PBS-TX (PBS containing 0.1% TX-100) for 10 min and then washed 3×, 5 min each with PBS-TX. Slides were finally drained and mounted in Mowiol, allowing 30 min for the Mowiol to set prior to examination by fluorescence microscopy (Olympus Provis), and image capture using a SmartCapture system.

### Immunostaining of larval brain squashes

Brains were dissected from second or third instar larvae in EBR as described above and fixed in 4% formaldehyde in PBS for 30 min at RT in a dissecting dish. Following this, the brains were transferred to a new well containing 45% acetic acid (in dH_2_O) for 3 min, then transferred to a drop (10–15 μl) of 60% acetic acid on a siliconized coverslip and squashed gently onto a poly-l-lysine slide. The slide was flash-frozen in liquid nitrogen and the coverslip was removed. The slide was placed in a Coplin jar with 100% ethanol at −20°C for 10 min. The brains were then permeabilized in PBS-Tx for 10 min at RT, washed twice in PBS, 5 min each, and then blocked in PBS + 3% BSA. After blocking, the slide was washed for 5 min in PBS-Tx at RT and then incubated 1–2 h at RT or overnight at 4°C in the primary antibody diluted in PBS + 0.3% BSA. The slide was washed 6× for 5 min each in PBS-Tx and the appropriate secondary antibody (diluted in 0.3% BSA in PBS) was applied for 1–2 h at RT. The slide was then washed 6×, 5 min each, in PBS-Tx. The brains were also stained with DAPI (0.1 μg/ml) included with the secondary antibody. Slides were finally drained and mounted in Mowiol, allowing 30 min for the Mowiol to set prior to examination by fluorescence microscopy (Olympus Provis fitted with a Hamamatsu ORCA II CCD camera), and image capture using a SmartCapture system.

Primary antibody and dilution used in immunostaining experiments was as follows: ubH2B @ 1:200 (Cell Signalling). Secondary antibody (Molecular Probes) used in immunostaining experiments was as follows: rabbit-Alexa Fluor-488 @ 1:500 dilution.

### Immunostaining of larval polytene chromosome spreads and whole mount larval salivary glands

The protocol from the Cavalli laboratory (Montpellier) was followed: http://www.igh.cnrs.fr/equip/cavalli/Lab%20Protocols/Immunostaining.pdf.

Salivary glands were dissected from third instar larvae in PBS-TX and fixed in 3.7% PFA in PBS-TX in a dissecting dish. For polytene chromosome spreads, salivary glands were then transferred to a drop of 3.7% PFA and 50% acetic acid on a siliconized coverslip. During incubation, salivary glands were fragmented and the remaining chitinuous structure of the pharynx removed. A poly-l-lysine slide was inverted on top of the coverslip and salivary glands were squashed. The chromosome spreads were quickly examined by phase microscopy and flash-frozen by dipping in liquid nitrogen. The coverslip was flicked off with a razor blade and slides were immersed immediately in a Coplin jar containing PBS 2× for 15 min. The slides with salivary glands were transferred to a blocking solution (3% BSA, 2% NP-40, 0.2% Tween-20, 10% non-fat dry milk in PBS) and blocked for 1 h at RT. After blocking, slides and salivary glands were incubated with primary antibody in blocking solution for 1 h at RT in a humid chamber. Slides and salivary glands were then rinsed in PBS and washed for 15 min first in wash solution 1 (300 mM NaCl, 0.2% NP-40, 0.2% Tween-20) and then in wash solution 2 (400 mM NaCl, 0.2% NP-40, 0.2% Tween-20). After washing, slides and salivary glands were rinsed in PBS and incubated with secondary antibodies in 0.2% NGS in blocking solution for 40 min at RT in humid chamber. After incubation slides and salivary glands were rinsed in PBS and washed for 15 min in wash solution 1 and wash solution 2, followed with rinsing in PBS. Slides and salivary glands were then stained for 10 min in 1 μg/ml in DAPI in PBS and washed in PBS for 5 min. Finally, slides were drained and mounted in Mowiol and salivary glands were mounted in 50% glycerol prior to examination by fluorescence microscope (Olympus Provis), and image capture using a SmartCapture system.

Primary antibodies and dilutions used in immunostaining experiments were as follows: ubH2B @ 1:50 (Medimabs), H3K4me3 @ 1:200 (Cell Signalling) and HP-1 @ 1:200. Secondary antibodies (Molecular Probes) used in immunostaining experiments were as follows: rabbit-Alexa Fluor-488, mouse-Alexa fluor-594, both used @ 1:500 dilution.

## RESULTS

### A second site non-complementation screen identifies a genetic interactor of *invadolysin*

Our previous studies focussed on the genetic characterization of *invadolysin* and on its localization in vertebrate cells (7,11). The original mitotic phenotype included abnormally condensed chromosomes, monopolar spindles, and an accumulation of nuclear envelope proteins. Despite detailed analysis of the mitotic phenotype, the molecular role of invadolysin was not apparent.

Second site non-complementation screens have served to identify interacting partners of various proteins (16–18). In *Drosophila*, failure to complement between heterozygous mutations at distinct loci (second site non-complementation) has been used to identify genes that have been shown in some cases to encode physically interacting proteins (19,20). We performed a second site non-complementation screen to identify genetic interactors of *invadolysin*, utilizing the DrosDel collection of molecularly-defined deficiencies that covers more than two-thirds of the genome (21). The transheterozygous progeny of heterozygous *invadolysin* and DrosDel deficiency stocks were scored on the basis of viability. The results of 110 crosses are shown in Table 1. Both the original *IX-14^1^* invadolysin allele and the DrosDel deficiency lines were maintained with balancer chromosomes, facilitating distinction of genetic classes amongst the progeny. The progeny lacking balancers represented the *IX-14^1^* mutation in the presence of a DrosDel deficiency (Df). Progeny were scored as viable, semi-lethal, or lethal–lethal representing a strong genetic interaction. Males and females were scored separately in order to determine if there were any gender-specific phenotypes.

**Table 1.** A second site non-complementation screen to identify genetic interactors of *invadolysin*

Df screen	Cytology	Interaction	Df screen	Cytology	Interaction
*Df(1)ED404*	1D2; 1E3	−	*Df(3L)ED212*	65A9; 65D5	-
*Df(1)ED6630*	3B1; 3C5	−	*Df(3L)ED4408*	66A22; 66C5	-
*Df(1)ED418*	5C7; 5E4	−	*Df(3L)ED4421*	66D12; 67B3	-
*Df(1)ED6957*	8B6; 8C13	−	*Df(3L)ED4475*	68C13; 69B4	-
*Df(1)ED429*	9D3; 9D3	−	*Df(3L)ED4483*	69A5; 69D3	-
*Df(1)ED7161*	11A1; 11B14	−	*Df(3L)ED215*	69B5; 69C4	-
*Df(1)ED7217*	12A9; 12C6	−	*Df(3L)ED4486*	69C4; 69F6	-
*Df(1)ED7294*	13B1; 13C3	−	*Df(3L)ED4502*	70A3; 70C10	-
*Df(1)ED447*	17C1; 17F1	−	*Df(3L)ED217*	70F4; 71E1	-
*Df(1)ED7413*	17D1; 17F1	−	*Df(3L)ED218*	71B1; 71E1	-
*Df(1)ED7441*	18A3; 18C2	−	*Df(3L)ED219*	71E1; 72F1	-
*Df(2L)ED2809*	21B1; 21B1	−	*Df(3L)ED220*	72D4; 72F1	-
*Df(2L)ED5878*	21B1; 21B3	−	*Df(3L)ED223*	73A1; 73D5	-
*Df(2L)ED62*	21D1; 21E2	−	*Df(3L)ED4674*	73B5; 73E5	-
*Df(2L)ED87*	21E2; 21E2	−	*Df(3L)ED4685*	73D5; 74E2	-
*Df(2L)ED123*	22B8; 22D4	−	*Df(3L)ED4710*	74D1; 75B11	-
*Df(2L)ED284*	25F2; 26A3	−	*Df(3L)ED224*	75B1; 75C6	-
*Df(2L)ED508*	28B1; 28C4	−	***Df(3L)ED225***	**75C1; 75D4**	**+**
*Df(2L)ED606*	28E1; 29C3	−	*Df(3L)ED4782*	75F2; 76A1	-
*Df(2L)ED629*	29B4; 29E4	−	*Df(3L)ED4786*	75F7; 76A5	-
*Df(2L)ED623*	29C1; 29E4	−	*Df(3L)ED4799*	76A1; 76B3	-
*Df(2L)ED647*	29E1; 29F5	−	*Df(3L)ED228*	76A1; 76D2	-
***Df(2L)ED695***	**30C5; 30E4**	**+/−**	*Df(3L)ED229*	76A1; 76E1	-
*Df(2L)ED784*	34A4; 34B6	−	*Df(3L)ED4858*	76D3; 77C1	-
*Df(2L)ED3*	35B2; 35D1	−	*Df(3L)ED4861*	76F1; 77E6	-
*Df(2L)ED1050*	35B8; 35D4	−	*Df(3L)ED4978*	78D5; 79A2	-
*Df(2L)ED1109*	36A3; 36A10	−	*Df(3L)ED230*	79C2; 80A4	-
*Df(2L)ED1203*	36F7; 37C5	−	*Df(3L)ED5017*	80A4; 80C2	-
*Df(2L)ED1303*	37E5; 38C6	−	*Df(3L)ED231*	80B1; 80C1	-
***Df(2R)ED1552***	**42A11; 42C7**	**+/−**	***Df(3R)ED5071***	**82A1; 82E4**	**+/-**
*Df(2R)ED1612*	42A13; 42E6	−	*Df(3R)ED5138*	82D5; 82F8	-
*Df(2R)ED1618*	42C3; 43A1	−	*Df(3R)ED5156*	82F8; 83A4	-
*Df(2R)ED1715*	43A4; 43F1	−	*Df(3R)ED5177*	83B4; 83B6	-
*Df(2R)ED1725*	43E4; 44B5	−	*Df(3R)ED5196*	83B9; 83D2	-
*Df(2R)ED1791*	44F7; 45F1	−	*Df(3R)ED5230*	84E6; 85A5	-
*Df(2R)ED2219*	47D6; 48B6	−	*Df(3R)ED5412*	85D15; 85E6	-
*Df(2R)ED2426*	51E2; 52B1	−	*Df(3R)ED5514*	86C7; 86E11	-
*Df(2R)ED2736*	53C6; 53F8	−	*Df(3R)ED5516*	86D8; 86E13	-
*Df(2R)ED3610*	54F1; 55C8	−	*Df(3R)ED5577*	86F9; 87B13	-
*Df(2R)ED3683*	55C2; 56C4	−	*Df(3R)ED5591*	87B7; 87C7	-
*Df(2R)ED3923*	57F6; 57F10	−	*Df(3R)ED5610*	87B11; 87D7	-
*Df(2R)ED4071*	60C8; 60E8	−	*Df(3R)ED5622*	87F10; 88A4	-
*Df(3L)ED4079*	61A5; 61B1	−	*Df(3R)ED5644*	88A4; 88C9	-
*Df(3L)ED201*	61B1; 61C1	−	*Df(3R)ED5705*	88E12; 89A5	-
*Df(3L)ED4177*	61C1; 61E2	−	*Df(3R)ED5780*	89E11; 90C1	-
*Df(3L)ED4191*	61C3; 62A2	−	*Df(3R)ED2*	91A5; 91F1	-
*Df(3L)ED207*	61C9; 62A6	−	*Df(3R)ED5911*	91C5; 91F8	-
*Df(3L)ED4284*	62B4; 62B12	−	*Df(3R)ED5942*	91F12; 92B3	-
*Df(3L)ED4287*	62B4; 62E5	−	*Df(3R)ED6076*	93E10; 94A1	-
*Df(3L)ED4288*	63A6; 63B7	−	*Df(3R)ED6096*	94B5; 94E7	-
*Df(3L)ED208*	63C1; 63F5	−	*Df(3R)ED6220*	96A7; 96C3	-
*Df(3L)ED4344*	63F5; 64B12	−	*Df(3R)ED6232*	96F10; 97D2	-
*Df(3L)ED4342*	64A12; 64B12	−	*Df(3R)ED6235*	97B9; 97D12	-
*Df(3L)ED210*	64B9; 64C13	−	*Df(3R)ED6265*	97E2; 98A7	-
*Df(3L)ED211*	65A9; 65B4	−	*Df(3R)ED6310*	98F12; 99B2	-

*IX-14^1^* mutants balanced over TM3 or TM6B were crossed to DrosDel deficiencies (e.g. Df) balanced over an appropriate balancer depending on the chromosome. The progeny were scored for the presence or absence of balancers (using dominant markers phenotypes associated with balancers). The transheterozygous flies with no balancer could be viable, semi-lethal or lethal based on the interaction between *IX-14* and genes deleted within the deficiency tested.

−: no interaction.

+/−: weak interaction, semi-lethal transheterozygous progeny.

+: strong interaction, lethal transheterozygous progeny.

As a result of the screening performed, one lethal (*Df(3L)ED225*) and three semi-lethal deficiencies (*Df(2L)ED695, Df(2R)ED1552* and *Df(3R)ED5071*) were identified. Attempts to identify interacting genes in the three semi-lethal deficiencies proved inconclusive, even though all smaller deficiencies and individual mutations available were tested. None of these deficiencies or mutations resulted in lethal or semi-lethal interaction with the *IX-14^1^* mutation, and therefore an unambiguous identification of the gene(s) responsible was not possible.

As the lethal *Df(3L)ED225* deficiency mapped to the left arm and the *invadolysin* mutation to the right arm of the third chromosome, the possibility that *Df(3L)ED225* merely uncovered the *invadolysin* gene could be eliminated. The *Df(3L)ED225* deficiency (located to the 75C-D region) completely deleted 23 genes and partially deleted an additional gene. Deficiency lines containing overlapping regions were tested for lethality with the *invadolysin* mutation. As the neighbouring *Df(3L)ED224* deficiency complemented *invadolysin*, genes in common were defined as non-interactors, leaving 17 genes as potential interactors. Smaller overlapping deficiencies (*Df(3L)H99* [viable] and *Df(3L)Exel6134* [lethal]) narrowed the number of possible interacting genes to 13. Four of the 13 genes were represented by mutant alleles in the Bloomington *Drosophila* stock centre (CG4166, CG13702, CG18231 and CG32197). Of these lines, only CG4166 was lethal in combination with the *IX-14^1^* mutation (Table 2).

**Table 2.** Secondary screen to identify *nonstop* as an interactor of *invadolysin*

Primary screen: Df	Secondary screen: Df or specific mutation	Cytology	Interaction with *IX-14*
*Df(2L)ED695*		30C5; 30E4	+/−
*Df(2R)ED1552*		42A11; 42C7	+/−
*Df(3R)ED5071*		82A1; 82E4	+/−
*Df(3L)ED225*		75C1; 75D4	**+**
	*Df(3L)H99*	75C1–2; 75C1-2	−
	*Df(3L)ED224*	75B1–75C6	−
	*Df(3L)Exel6134*	75C7; 75D4-13	**+**
	*CG32197 (Met75Ca)*	75C7	−
	*CG13702 (Aicr2)*	75D1	−
	*CG18231*	75D4	−
	*CG4166 (not)*	75D4	**+**

During the primary screen (Table 1), one lethal [*Df(3L)ED225*] and three semi-lethal [*Df(2L)ED695, Df(2R)ED1552* and *Df(3R)ED5071*] deficiencies were identified. The potential interactors of *IX-14^1^* from the pool of 23 genes uncovered by *Df(3L)ED225* were narrowed down by crossing *IX-14^1^* to the overlapping deficiency *Df(3L)ED224*. Six genes were eliminated as candidate interactors as *Df(3L)ED224* complemented *IX-14^1^*. Deficiencies *Df(3L)H99* and *Df(3L)Exel6134* were tested to further narrow down the number of potential interacting genes. Individual mutant alleles within *Df(3L)Exel6134* were tested for lethality and a mutation in the gene CG4166 was found to be lethal in combination with *IX-14^1^* (transheterozygous animals die as pupae). CG4166 encodes nonstop (not).

−: no interaction.

+/−: weak interaction, semi-lethal transheterozygous progeny.

+: strong interaction, lethal transheterozygous progeny.

### Identification of *nonstop* as a genetic interactor of invadolysin

CG4166 was previously named *nonstop* or *not* (22). *IX-14^1^*/*not^1^* transheterozygotes were 100% lethal and died prior to eclosion from pupal cases, demonstrating the genetic interaction between *invadolysin* and *nonstop*.

**Figure 1.**
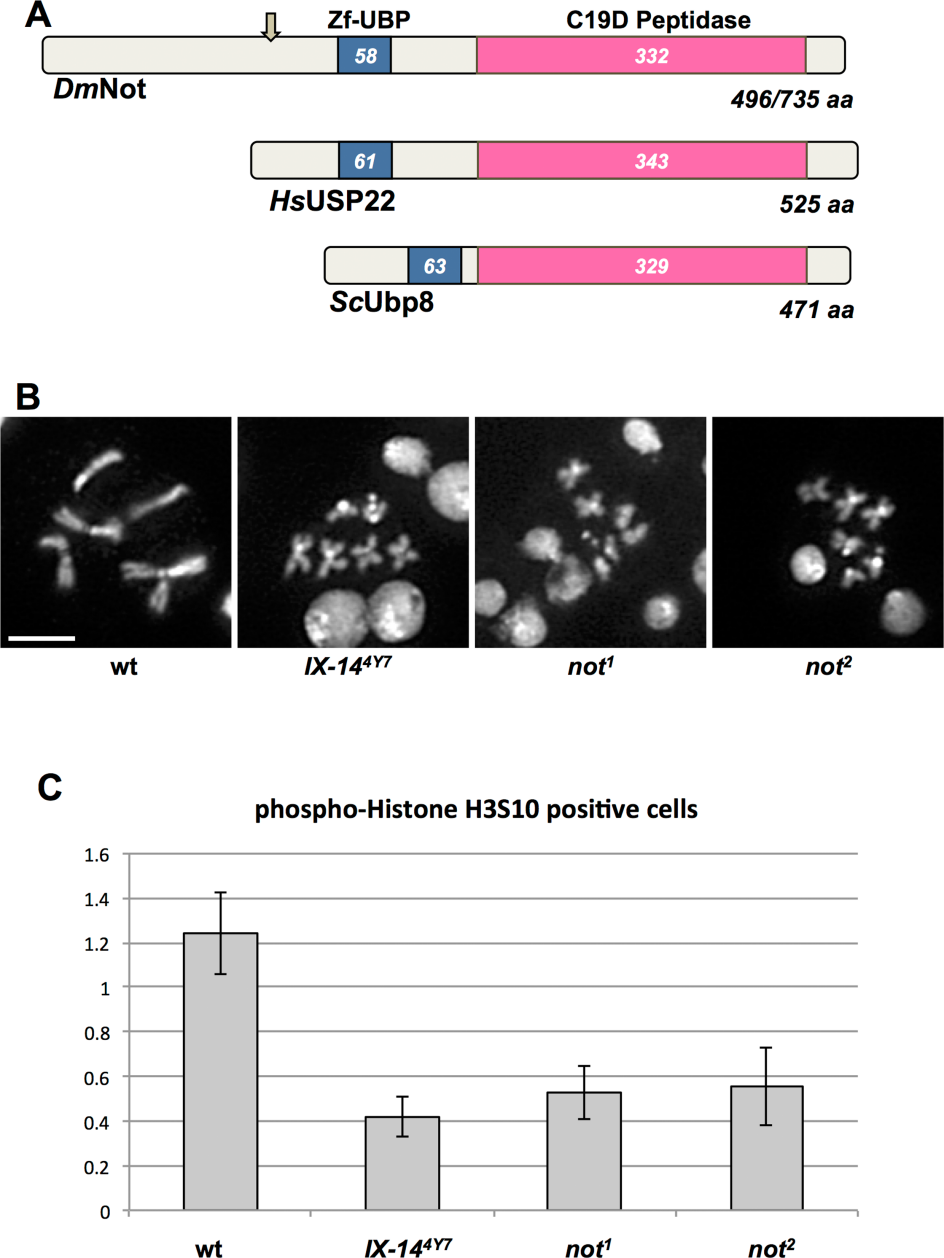
*nonstop* interacts genetically with *invadolysin* and exhibits similar phenotypes to *invadolysin* mutants. (**A**) *Drosophila* Nonstop is a highly conserved protein belonging to the C19 class of DUB cysteine proteases, with a peptidase domain at the C-terminus, and a zinc finger motif in the central region. *Drosophila* nonstop exhibits homology to UBP8 from *Saccharomyces cerevisiae* and human USP22. The lengths of the proteins and locations of the relevant domains are shown. (**B**) *invadolysin* and *nonstop* mutants exhibit similar mitotic chromosome phenotypes after staining with DAPI. Both *invadolysin* and *nonstop* mitotic chromosomes are hypercondensed in length, yet fuzzy in appearance (scale bar = 5 μm). (**C**) The mitotic index was decreased in both *invadolysin* and *nonstop* mutant brains. Cells were counted from 10 fields each from three brains for all the genotypes shown. The mitotic index (following immunostaining for phospho-histone H3S10) of mutants (*invadolysin* and *nonstop*) was significantly lower than in wild-type neuroblasts. The p-values are: *IX-14^4y7^P* = 0.002, *not^1^ P* = 0.005 and *not^2^ P* = 0.01.

*nonstop* is a homozygous pupal lethal mutation so named because of its ‘nonstop’ axon guidance phenotype, as it is required for the correct migration of the R1–R6 axons (22,23). The *nonstop* gene encodes a DUB, or deubiquitinating enzyme—a ubiquitin specific protease (23). Nonstop is described in *Drosophila* as either a 496 or a 735 amino acid (differing in the length of the N-terminus) conserved protein with a more N-terminal zinc finger motif and a C-terminal C19D cysteine peptidase domain (Figure 1A). *Drosophila* nonstop, human USP22 and *Saccharomyces* Ubp8 share significant homology. By virtue of their ability to regulate protein ubiquitination in a target-specific manner, ubiquitin specific proteases play roles in protein degradation by the proteasome and in other ubiquitin-dependent processes (24).

### Similar phenotypes in *invadolysin* and *nonstop* mutants

The original phenotype described for the *invadolysin* mutant larval neuroblasts was one of abnormally condensed chromosomes (7,25). While mitotic chromosomes were hypercondensed in length, they appeared fuzzy in overall appearance. We hypothesized that the condensation defect was due to an underlying structural defect rather than to a mitotic arrest, since allowing more time in mitosis with colchicine treatment (thereby depolymerizing microtubules) failed to rescue the fuzziness observed in *invadolysin* chromosomes (7). Interestingly, we discovered that two different *not* alleles (*not^1^* and *not^2^*) also had aberrantly condensed chromosomes, which were similar in appearance to chromosomes in the *invadolysin* mutant (Figure 1B). Heterozygous *invadolysin* and *nonstop* larvae were completely viable, and the mitotic chromosomes of heterozygous neuroblasts appeared normal.

The mitotic index (the percentage of cells in mitosis) reflects the extent of cell proliferation. The mitotic index for both *invadolysin* and *nonstop* mutant neuroblasts was 3-fold lower compared to wild type (Figure 1C). The mitotic index of all mutant alleles analysed following staining for phospho-histone H3S10 was significantly different from wild type based on a one-way ANOVA (*P*-values given in the figure legend). Thus, we conclude that cell proliferation was disrupted in both *invadolysin* and *nonstop* mutants. Interestingly, human USP22 has been linked to suppression of cell proliferation in cancer (26). We conclude that *nonstop* and *invadolysin* mutants share numerous phenotypes, suggesting that these two proteases participate in the same or closely related cellular pathways.

### *Invadolysin* and *nonstop* mutants, and *IX-14^1^*/*not^1^* transheterozygous larvae accumulate specific histone modifications

**Figure 2.**
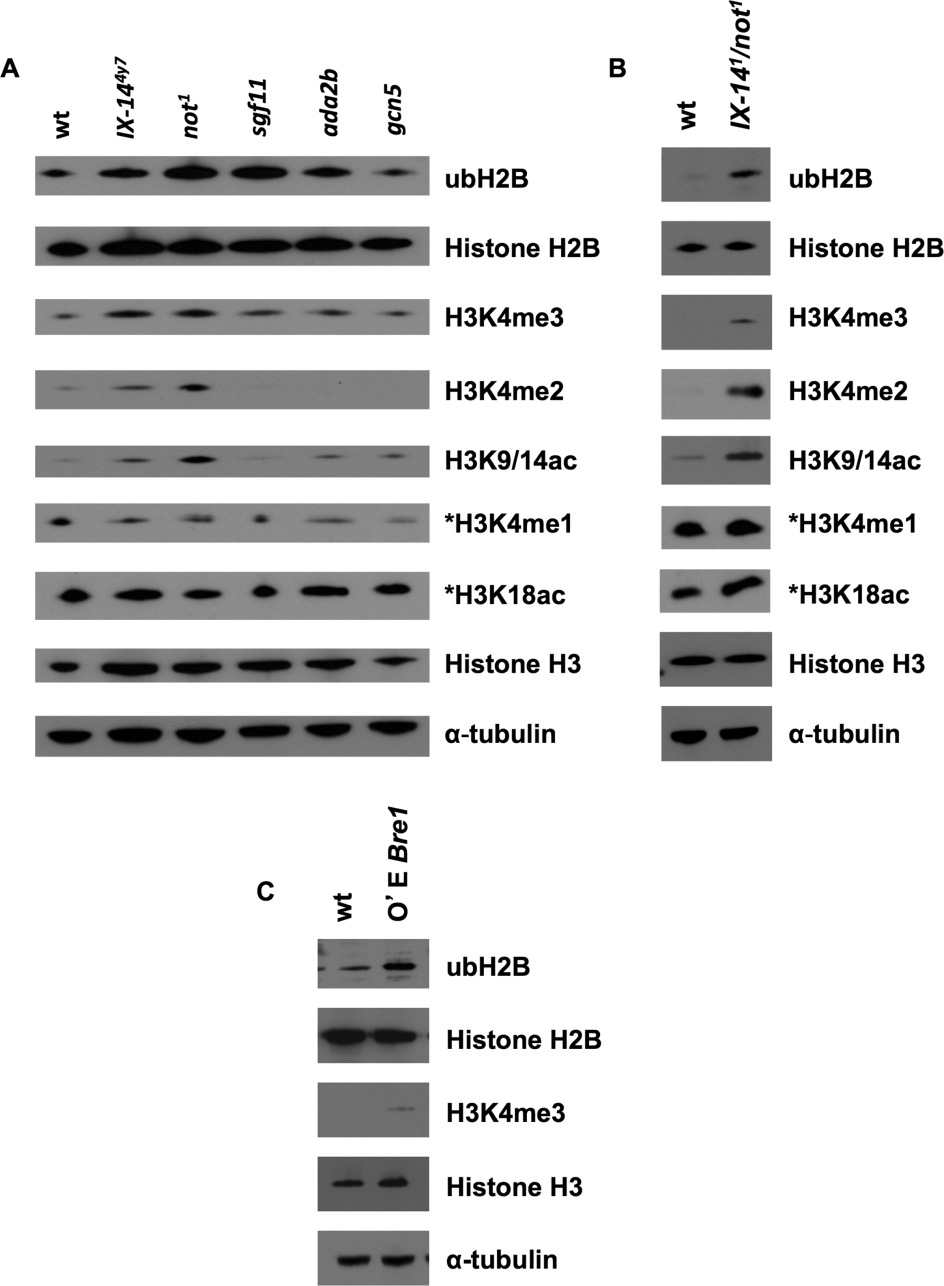
*invadolysin* and *nonstop* homozygous, and *IX-14^1^/not^1^* transheterozygous third instar larvae accumulate similar histone modifications. Over-expression of the Bre1 ubiquitin ligase phenocopies this effect. (**A**) Immunoblotting of *invadolysin, nonstop, sgf11, ada2b* and *gcn5* homozygous third instar larval extracts using various antibodies revealed changes to histone modification. Mono-ubiquitinated histone H2B, di- and tri-methylated histone H3 at lysine 4, and acetylated H3K9/14 were increased in *invadolysin* and *nonstop* larval protein extracts. (**B**) Immunoblotting of *IX-14^1^/not^1^* transheterozygous third instar larval extracts using the same antibodies as in (A) revealed a similar accumulation of mono-ubiquitinated histone H2B, di- and tri-methylated histone H3 at lysine 4, and acetylated H3K9/14 in transheterozygous larval protein extracts as well. The levels of non-SAGA dependent modified histones in (A) and (B) remain unchanged (*H3K4me1 and *H3K18ac). (**C**) Immunoblotting of dBre1 overexpressing (using an actin-Gal4 driver) third instar larval extracts revealed the accumulation of ubH2B and H3K4me3. Loading controls for panels (A)–(C) are represented by histone H2B, histone H3 and α-tubulin.

Immunoblotting individual *invadolysin* and *nonstop* mutant larval extracts for ubiquitin revealed the accumulation of ubiquitinated proteins at ∼200, 55 and 29 kDa (data not shown) as previously reported for *nonstop* mutants (23). Further studies on *nonstop* mutants confirmed that the 29 kDa protein was mono-ubiquitinated histone H2B (ubH2B) (27). We observed a similar accumulation of ubH2B in *IX-14* homozygous, *not^1^* homozygous and *IX-14^1^*/*not^1^* transheterozygous larvae (Figure 2A and B). Ubiquitination of H2B has been shown to silence (28) or activate (29) transcription (30) by influencing the di- and tri-methylation of histone H3 on lysine 4 and 79 by the COMPASS complex and Dot1 methyltransferase (31). We demonstrate here that homozygous *invadolysin, nonstop* and transheterozygous *IX-14^1^*/*not^1^* larval extracts accumulate H3K4me2 and H3K4me3, as well as ubH2B (Figure 2A-B). Recent findings confirmed that acetylation of histone H3 at lysine 9 and 14 is dependent on di- and tri-methylation of histone H3 at lysine 4 (32,33). The level of K3K9/14ac was also increased in *invadolysin, nonstop* and *IX-14^1^*/*not^1^* transheterozygotes (Figure 2A and B). In contrast, levels of non-SAGA dependent modified histones (H3K4me1 and H3K18ac) (34,35) were unchanged in homozygous mutant and transheterozygous extracts (Figure 2A and B). Quantitation of the immunoblots shown in Figure 2 is presented in Supplementary Figure S1, and demonstrates the increase in ubH2B, H3K4me2 and H3K4me3 while the unmodified H2B and H3 histones remain relatively constant. Neither heterozygous *invadolysin*, nor heterozygous *nonstop* larval extracts showed accumulation of modified histones. Therefore, the change in histone modification is associated with the homozygous mutant chromosome phenotype.

We previously demonstrated that *invadolysin* alleles suppressed position effect variegation in *wm4* flies, as shown by an increase in expression of the white reporter gene and the likely loosening of chromatin. This observation is consistent with a role for the wild-type invadolysin gene in chromatin compaction (7). As histone ‘trans-tail’ cross-talk is considered to be important during the early stages of transcription (36,37), we suggest that the specific effects on histone post-translational modification might be manifest as effects on gene expression—as observed in the position effect variegation assay.

### Over-expression of H2B ubiquitin ligase Bre1 phenocopies the hypercondensed mitotic chromosome phenotype and modified histone accumulation

**Figure 3.**
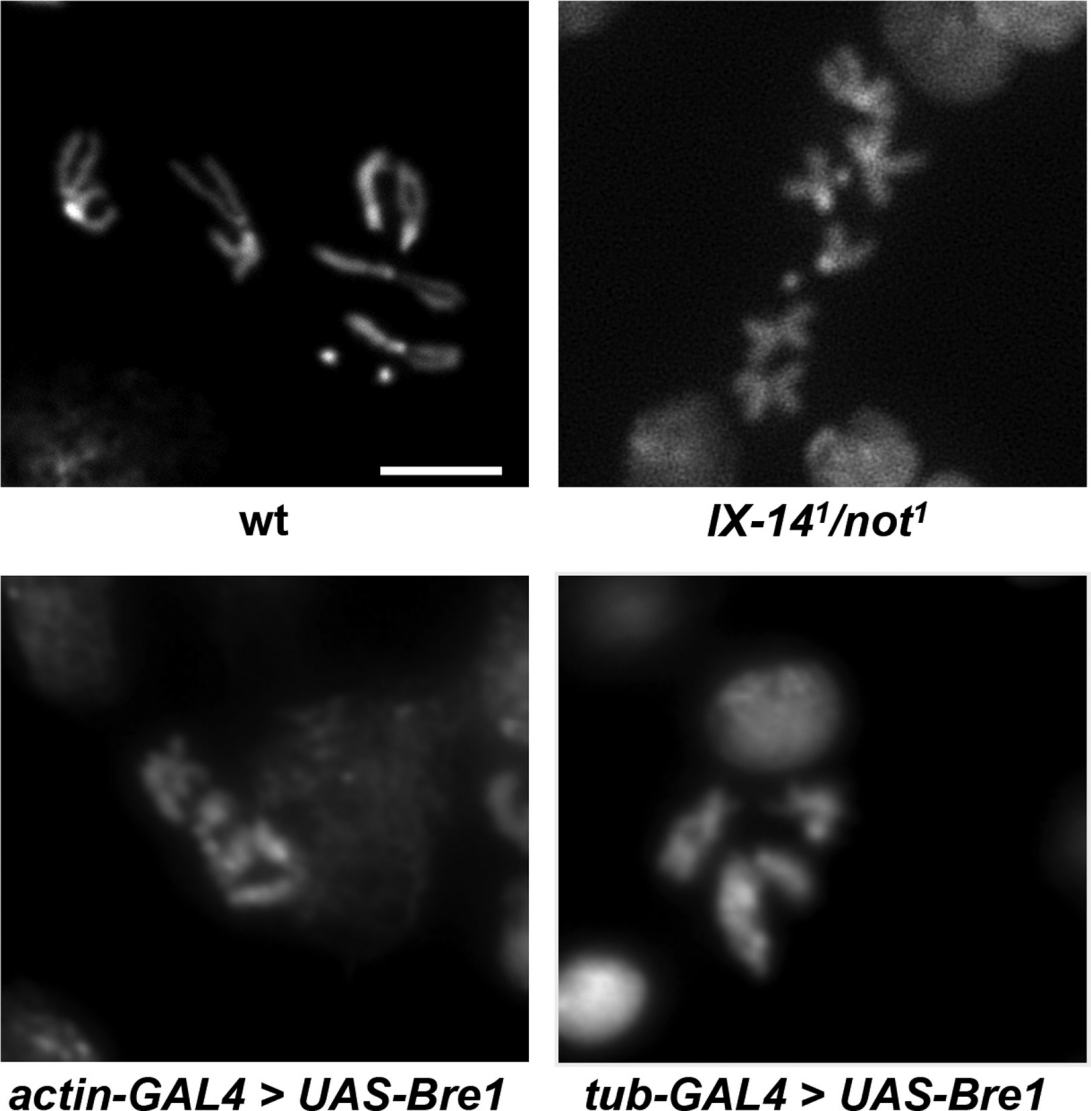
Mitotic chromosomes from *IX-14^1^/not^1^* transheterozygous and Bre1 overexpression neuroblasts are abnormal in appearance. *IX-14^1^/not^1^* transheterozygous neuroblasts exhibit similar mitotic chromosome phenotypes to *invadolysin* and *nonstop* homozygous neuroblasts after staining with DAPI. Mitotic chromosomes from *IX-14^1^/not^1^* transheterozygous neuroblasts are hypercondensed in length, yet fuzzy in appearance. The chromosomes following overexpression of the Bre1 ubiquitin ligase with either an actin-GAL4 or tubulin-GAL4 driver are also hypercondensed (images are shown at the same magnification). Scale bar = 5 μm.

The identification of nonstop as a DUB targeting H2B implies a corresponding E3 ligase that ubiquitinates H2B. Bre1 is known to ubiquitinate H2B in yeast (38). Therefore, we hypothesized that over-expression of Bre1 might result in increased mono-ubiquitination of H2B, and possibly phenocopy the chromosome condensation defects observed in *invadolysin* or *nonstop* mutants. When *Drosophila* Bre1 (dBre1) was over-expressed ubiquitously (using an *actin-GAL4* driver to express *UAS-bre1*), accumulation of both ubH2B and H3K4me3 was detected in larval extracts (Figure 2C). In addition, the neuroblast chromosomes appeared hypercondensed after over-expression of Bre1 (with either an *actin-* or a *tubulin-GAL4*) compared to *IX-14^1^*/*not^1^* transheterozygotes (Figure 3). This strongly suggested that the hypercondensed appearance of chromosomes was a consequence, directly or indirectly, of the accumulation of specific histone modiifcations.

### Polytene chromosomes appear abnormal in *invadolysin, nonstop* and transheterozygous mutants

**Figure 4.**
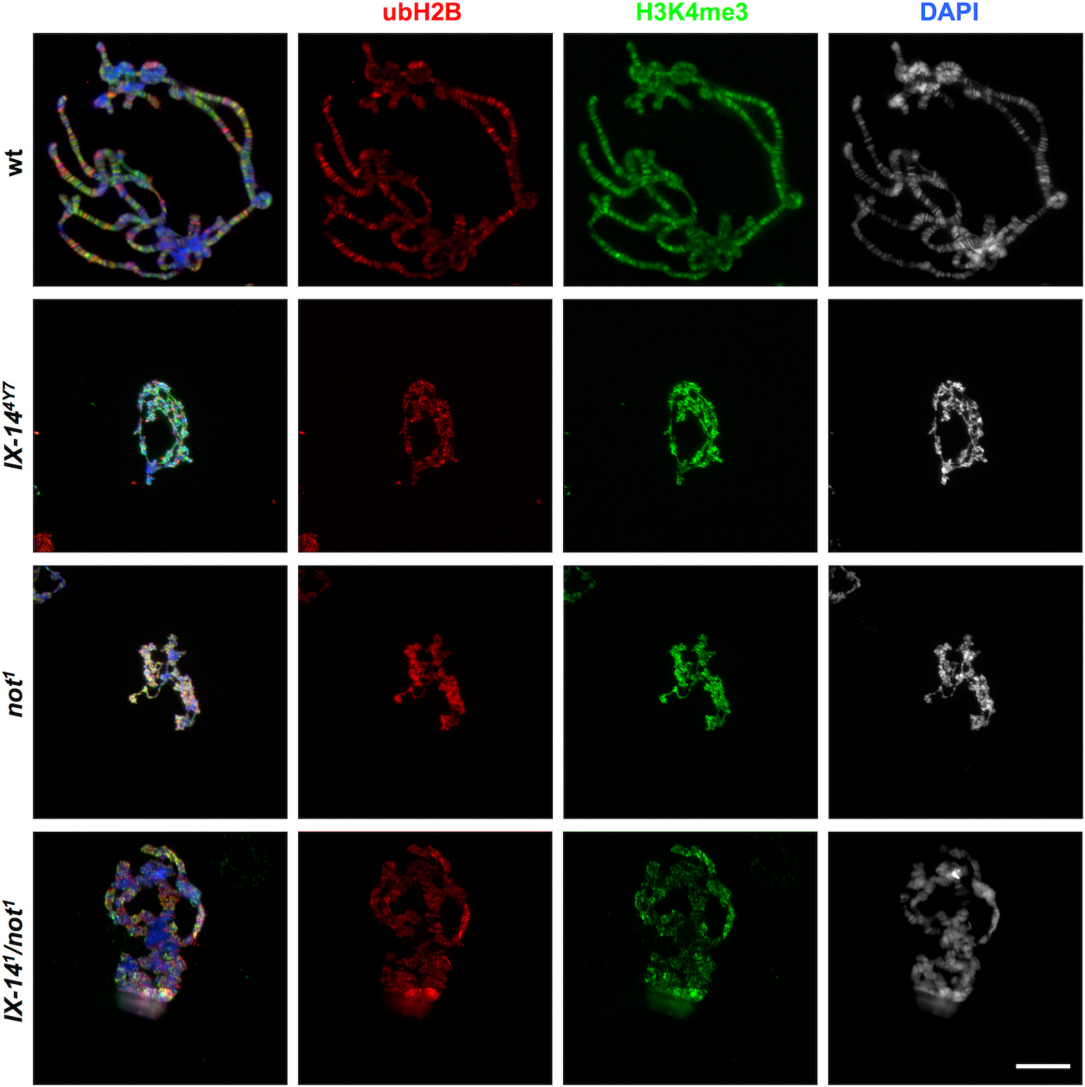
Polytene chromosomes from *invadolysin, nonstop* and *IX-14^1^/not^1^* transheteroygous mutants are abnormal in appearance. Salivary glands from third instar larvae were dissected and squashed. Polytene chromosomes were stained for ubH2B (red), H3K4me3 (green) and DAPI (blue). *invadolysin, nonstop* and *IX-14^1^/not^1^* transheterozygous polytene chromosomes were abnormal and had lost the characteristic banding pattern. Scale bar = 20 μm.

The salivary glands of *Drosophila melanogaster* contain giant, multi-stranded polytene chromosomes that are formed by repeated rounds of DNA replication in the absence of mitosis, with the 1000–2000 chromatids aligning in register and the creation of a chromocentre by the fusion of the centromeres. We reported previously that *invadolysin* mutant salivary gland chromosomes were structurally defective (7). In order to determine whether polytene chromosomes in *nonstop* were also similarly defective, we examined polytene chromosomes from salivary glands after staining for DNA, ubH2B and H3K4me3 (Figure 4). Indeed both *invadolysin* and *nonstop* mutants showed poorly replicated and banded polytene chromosomes. The chromosome arms appeared non-distinct, with an ill-defined chromocentre. Immunostaining with HP-1 (heterochromatin protein-1) antibody revealed the presence of chromocentre and telomeres, which appeared as smaller foci in homozygous *invadolysin, nonstop* and *IX-14^1^*/*not^1^* transheterozygous polytenes (data not shown). In spite of these morphological differences, staining of the chromatin demonstrated readily detectable levels of ubH2B and H3K4me3 in wild type and mutant chromosomes.

### ubH2B and H3K4me3 modification show different patterns of localization in polytene chromosomes

Due to the presence of a reproducible banding pattern, *Drosophila* polytene chromosomes have been used to investigate chromatin structure at cytological and molecular levels. The more loosely-condensed ‘interbands’ generally represent regions of greater transcriptional activity (e.g. euchromatin), while the compact ‘bands’ are more representative of heterochromatic character. In contrast, the chromocentre is comprised of constitutive heterochromatin (39).

**Figure 5.**
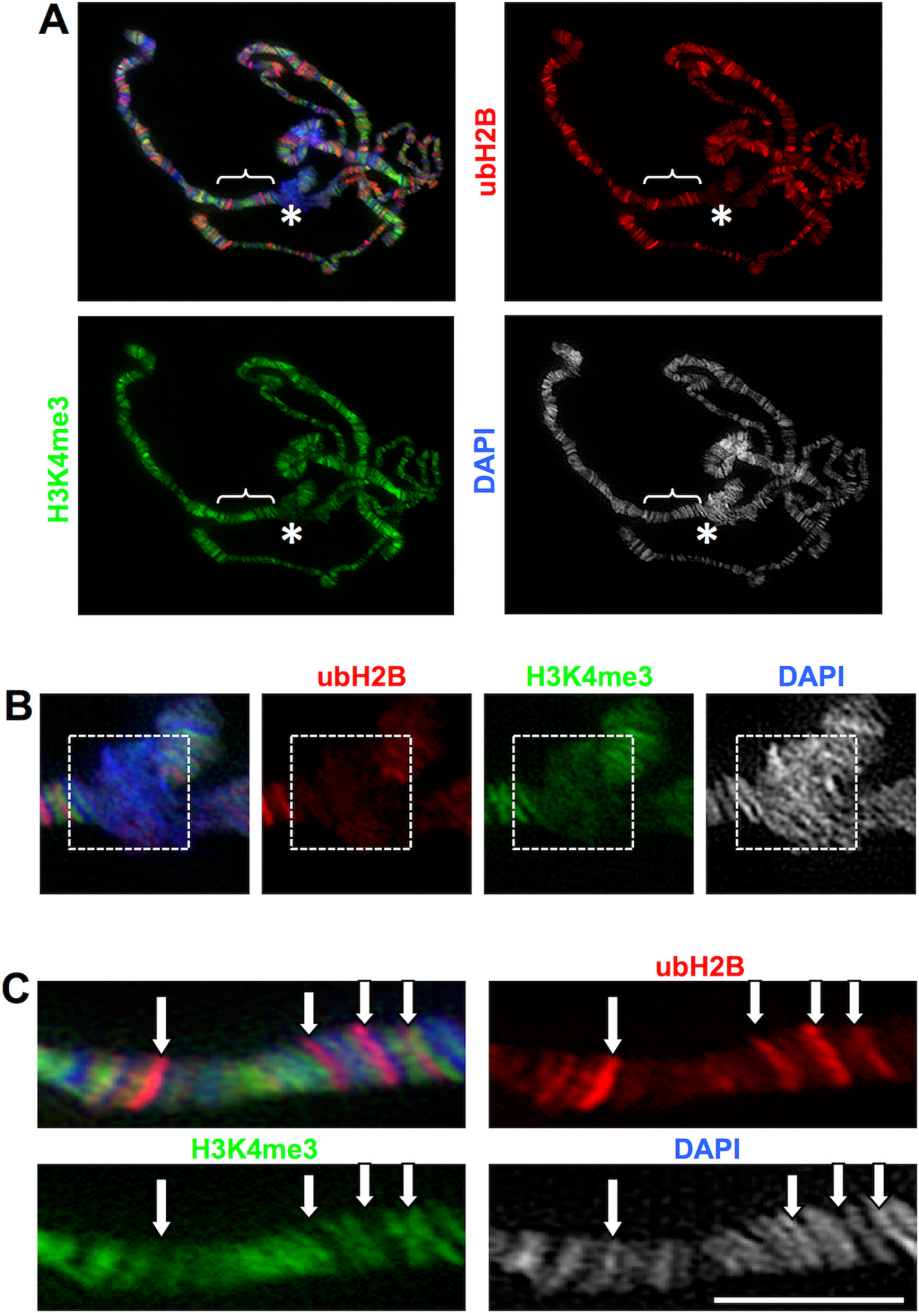
ubH2B is predominantly associated with interbands in polytene chromosomes and does not co-localize with H3K4me3. (**A**) Wild-type third instar larval salivary glands were fixed and squashed, and polytene chromosomes immunostained for ubH2B (red), H3K4me3 (green) and DAPI (blue). The chromocentre (asterisk) is enlarged in (B), while the bracketed section of a chromosome arm is enlarged in (C). (**B**) Immunostaining of the polytene chromocentre (primarily heterochromatic) revealed decreased amounts of ubH2B and H3K4me3 (white dotted box). (**C**) The ubH2B and H3K4me3 modifications do not appear to co-localize with one another. Scale bar = 20 μm.

A striking banding pattern for both ubH2B and H3K4me3 was apparent along wild type polytene chromosomes (Figure 5). ubH2B and H3K4me3 were noticeably lower at the chromocentre, suggesting that these two modifications were not as frequently associated with heterochromatin (Figure 5B). Paradoxically, ubH2B and H3K4me3 showed a general lack of co-localization on chromosome arms (Figure 5C, Supplementary Figures S2 and S3). We performed quantitative pixel analysis (using ImageJ) of the chromocentre and three independent segments of polytene chromosomes for the patterns of ubH2B and H3K4me3 (Supplementary Figure S4). This scanning analysis confirmed a general lack of co-localization between these modifications in wild type polytene chromosomes.

### Invadolysin and the SAGA complex

The human and yeast homologues of *nonstop* (*USP22* and *ubp8*) are components of the SAGA transcriptional co-activator complex which is highly conserved amongst eukaryotes (40). The SAGA complex (comprised of approximately 20 different protein subunits) contains two well-characterized enzymatic activities: the deubiquitinating “DUB” activity of Nonstop in conjunction with the Sgf11 subunit, and a histone acetyltransferase “HAT” module utilizing the Gcn5 and Ada2B components (27). As *invadolysin* and *nonstop* exhibit a genetic interaction, we hypothesized that invadolysin may also interact with other subunits of the SAGA complex.

**Figure 6.**
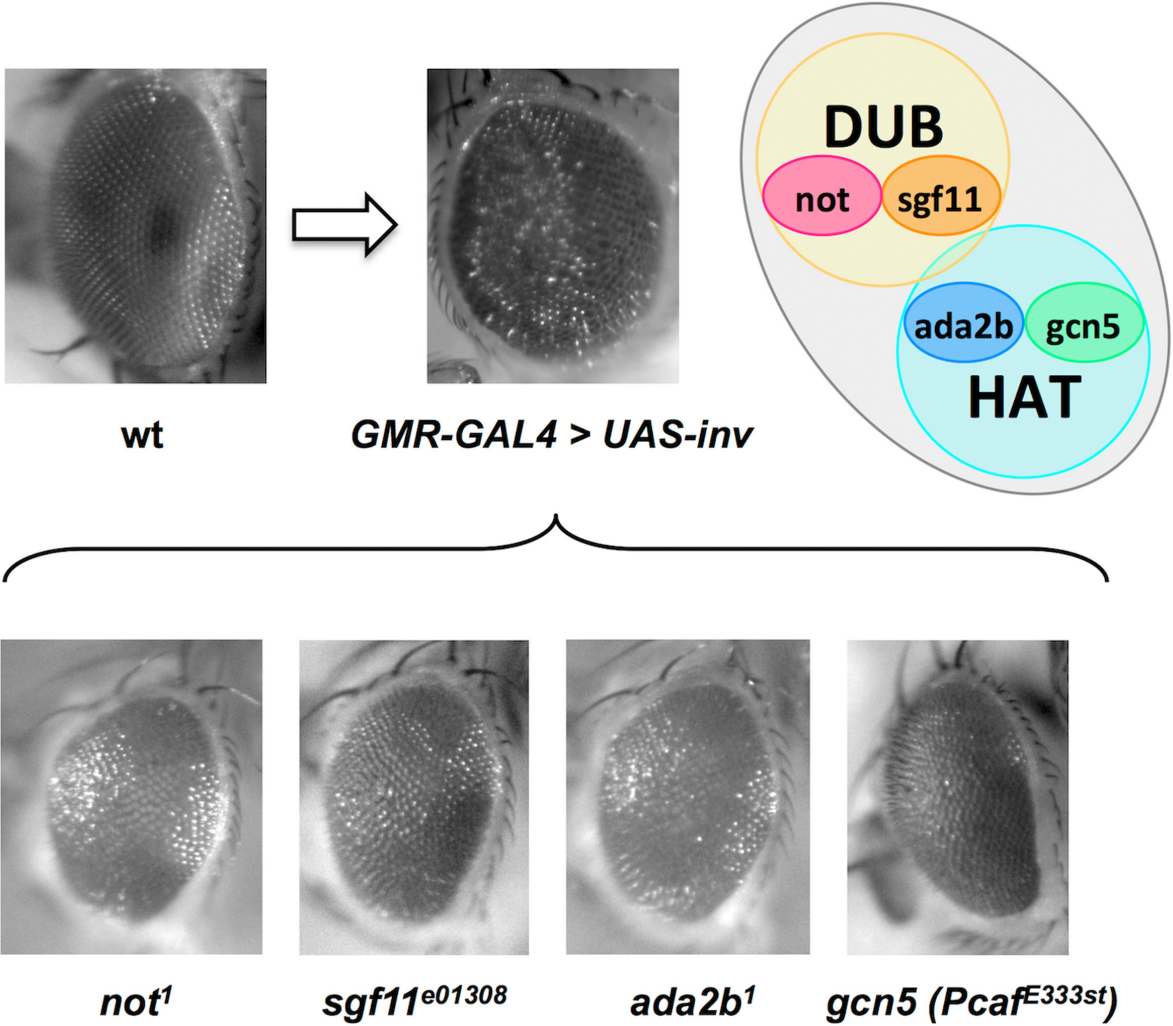
Additional subunits of the SAGA complex show genetic interaction with invadolysin, in an invadolysin-induced rough-eye modification assay. Images show *Drosophila* compound eyes. (**A**) A rough-eye phenotype results when the expression of UAS-invadolysin is induced in the eye by a GMR-GAL4 driver (compare with the wild-type eye). (**B**) The invadolysin-induced rough-eye phenotype is suppressed by mutations of subunits of both DUB (*not* and *sgf11*) and HAT (*gcn5* and *ada2b*) modules of the SAGA complex.

In order to determine whether invadolysin interacted genetically with other SAGA subunits, we screened for modification of an invadolysin over-expression phenotype. Over-expression of invadolysin in the eye (*GMR-GAL4* driving a *UAS-invadolysin* transgene) resulted in a rough eye phenotype (Figure 6). Mutant alleles of DUB (*nonstop, sgf11*), and HAT subunits (*gcn5, ada2b*) were crossed to flies over-expressing *invadolysin* in the adult eye. This screen independently confirmed the genetic interaction of *invadolysin* with *nonstop* as both *not^1^* and *not^2^* alleles suppressed the *invadolysin*-induced rough eye phenotype. As *sgf11, ada2b* and *gcn5* also similarly suppressed the invadolysin-induced rough eye phenotype, we conclude that *invadolysin* interacts genetically with multiple SAGA subunits (Figure 6). All genetic suppressions observed were moderate (not full)—nonetheless a clear effect on eye roughness was apparent during screening at the level of bright field microscopy (albeit difficult to capture digitally). Through trial and error, we have determined that grey-scale images demonstrated the suppression phenotype more clearly than colour depiction. This assay is inherently non-quantitative, therefore we use the modification of rough eye phenotype as only one line of experimentation in our analyses.

The SAGA complex exhibits both DUB and HAT activity, with the HAT activity directed to H3K9/14. In this context, it is worth noting that we also observe an increase in the level of H3K9/14ac in homozygous *invadolysin* and *nonstop*, and transheterozygous *IX-14^1^*/*not^1^* larval extracts (Figure 2A and B). Taken together, our data suggest that invadolysin may act to regulate both the DUB and HAT activities of the SAGA complex—potentially by playing a role in the assembly or activity of SAGA.

### Accumulation of ubH2B in the cytoplasm of *IX-14^1^*/*not^1^* salivary glands

**Figure 7.**
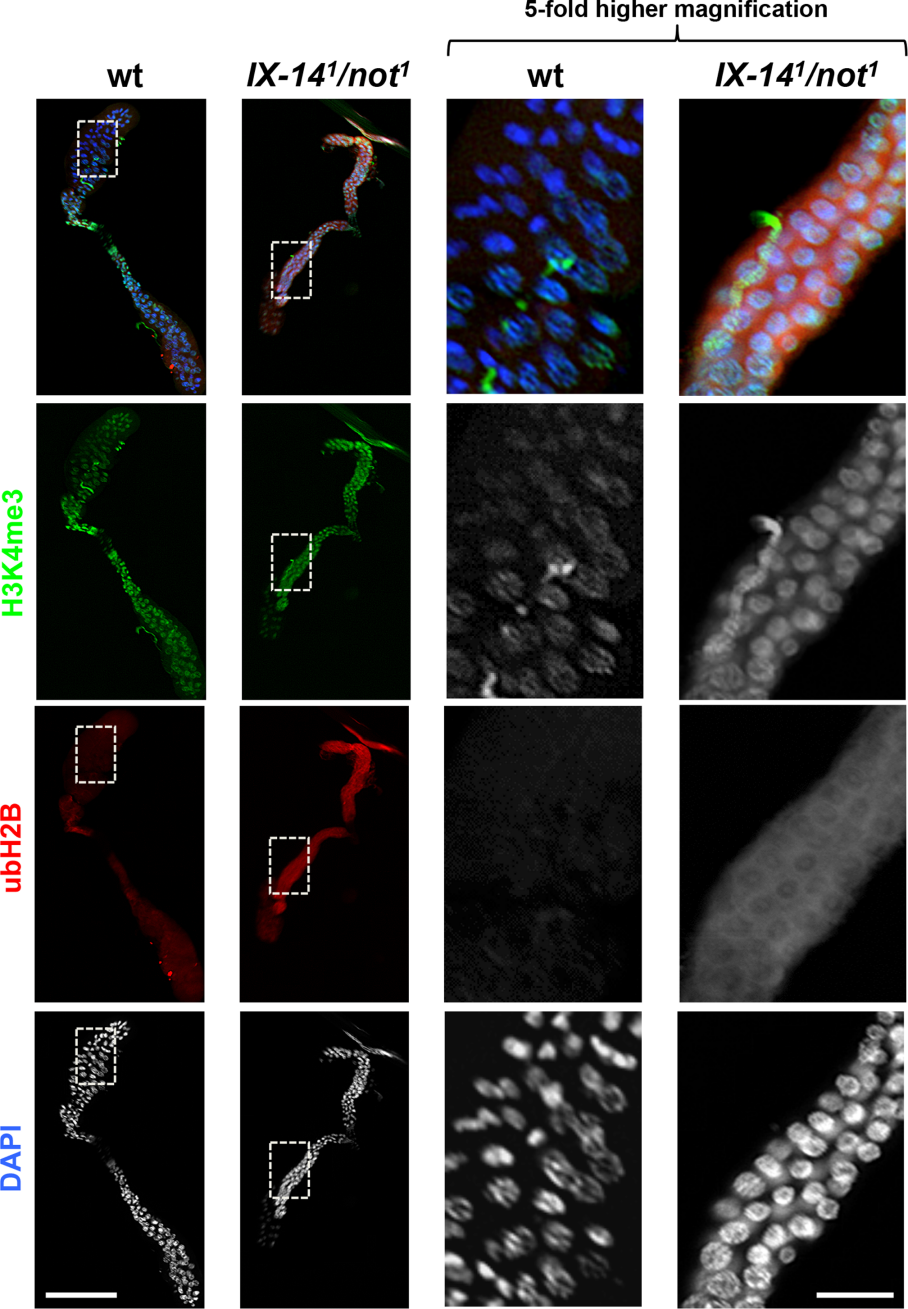
Whole mount immunostaining of *IX-14^1^/not^1^* transheterozygous salivary glands revealed an unexpected accumulation of ubH2B in the cytoplasm. Salivary glands from wild type and *IX-14^1^/not^1^* transheterozygous third instar larvae were dissected, fixed and immunostained for H3K4me3 (green), ubH2B (red), and DAPI (blue). As observed in wild type salivary glands, H3K4me3 was present in the nuclei of *IX-14^1^/not^1^* transheterozygous salivary glands. Conversely, ubH2B appears to accumulate in the cytoplasm of *IX-14^1^/not^1^* transheterozygous salivary glands. Scale bar for the low magnification images on the left half of the figure = 600 μm. Scale bar for the 5-fold higher magnification images on the right half of the figure = 120 μm.

Mitotic and polytene chromosomes were cytologically abnormal in both *invadolysin* and *nonstop* mutants, and ubH2B and H3K4me3 were elevated in homozygous *invadolysin* and *nonstop*, and transheterozygous *IX-14^1^*/*not^1^* larval extracts. These observations suggest that the accumulation of these modified histones may accompany the appearance of abnormally structured chromosomes. We therefore examined whole mount *IX-14^1^*/*not^1^* transheterozygous salivary glands for DNA, ubH2B and H3K4me3 (Figure 7). Nuclei of *IX-14^1^*/*not^1^* glands appeared smaller in whole mounts, corroborating the observation of smaller chromosomes in polytene squashes. As expected, H3K4me3 accumulates in the nuclei of *IX-14^1^*/*not^1^* transheterozygous salivary glands (2^nd^ row). Surprisingly, however, ubH2B accumulates prominently in the cytoplasm of *IX-14^1^*/*not^1^* transheterozygous salivary glands (third row).

As the salivary glands represent a polyploid, non-mitotically active tissue, we also examined diploid *invadolysin* and *nonstop* mutant, and *IX-14^1^*/*not^1^* transheterozygous neuroblasts for ubH2B (Supplementary Figure S5). While we observed staining throughout interphase neuroblasts, we also observed a striking association of ubH2B with mitotic chromosomes, particularly evident in the *nonstop* mutant. The unanticipated cytoplasmic localization of ubH2B begs the question of why this modified histone accumulates in the cytoplasm, and what downstream consequence(s) this localization may have.

## DISCUSSION

### Identification of *nonstop* as a genetic interactor of *invadolysin*

We performed this investigation with the goal of identifying genetic interactors of *invadolysin* to further decipher the cellular and molecular function(s) of this novel metalloprotease. Second site non-complementation screening in *Drosophila* identified the *nonstop* ubiquitin protease as one such interactor. This discovery is significant not least because proteases frequently act in cascades or pathways to carry out their physiological functions.

### Phenotypic comparison of *invadolysin* and *nonstop* mutants

Consistent with the *nonstop* gene encoding a DUB, we detected accumulation of ubH2B in *nonstop* as well as in *invadolysin* mutant larvae. The observation that *IX-14^1^*/*not^1^* transheterozygous larvae also showed an accumulation of ubH2B suggests that the two proteins may be functioning in the same or related pathways to regulate an essential aspect of chromatin packaging. In support of this hypothesis, the chromosome condensation phenotype observed in *nonstop* mitotic cells was morphologically similar to that observed in *invadolysin* neuroblasts. The polytene chromosomes in salivary glands also shared similar mutant phenotypes. However, the extent of mitotic defects was less pronounced in *nonstop* mutants compared to *invadolysin* mutants (e.g. mitotic spindles appeared normal, data not shown), suggesting that *invadolysin* might be acting upstream in the pathway and hence resulting in more severe defects when mutated.

### Regulation of histone modification by invadolysin

Ubiquitination of H2B promotes activation of transcription as well as telomeric silencing (28,30,40). The yeast homologue of *nonstop* (*ubp8*) has been shown to deubiquitinate histone H2B and thereby regulate transcription (40). Ubp8, like nonstop, is a member of the SAGA complex (41,42). Nonstop (along with the SAGA complex) is involved in the control of axon guidance in *Drosophila* via histone deubiquitination (23,27). We hypothesize that invadolysin together with nonstop might be involved in the regulation of transcription by deubiquitinating H2B, as ubH2B accumulates in extracts of *IX-14^1^*/*not^1^* transheterozygous larvae. Intriguingly, we also observed the accumulation of H3K4me3, H3K4me2 and H3K9/14ac modifications, consistent with previously suggested ‘trans-tail’ cross-talk between these histone modifications (31,43).

While the SAGA complex contains two well-characterized enzymatic modules that deubiquitinate and acetylate histones, the functions of several subunits in the SAGA complex are still unknown. It is unclear how the members of SAGA interact to regulate or coordinate the DUB and HAT activities of this complex, or whether cooperative or antagonistic interactions between these modules occur. Based on the genetic interaction of *invadolysin* with both DUB and HAT components of the SAGA complex (*nonstop/Sgf11* and *Gcn5/Ada2B*), we propose that invadolysin may play a role in the regulation of both activities and thus contribute a crucial role during the processes of chromatin remodelling and transcriptional activation (44,45).

The DUB activity of nonstop in the H2B ubiquitination cycle is balanced by an E3 ubiquitin ligase, identified in yeast as Bre1 (38,46). We demonstrate herein that over-expression of dBre1 ligase results in ubH2B accumulation and a similar chromosomal phenotype to that observed in *invadolysin* and *nonstop* mutants. Our observations support the hypothesis that the aberrant chromosome phenotype is a consequence of ubH2B accumulation. ubH2B can influence transcription by affecting H3K4me3 accumulation (34), which was greatly increased in both *IX-14^1^*/*not^1^* transheterozygous and *Bre1* over-expressing larval extracts. H3K4me3 is linked to transcriptional activation (47), consistent with our observation that *invadolysin* alleles act as suppressors of position effect variegation (7). Hence, we conclude that both *invadolysin* and *nonstop* mutants may show abnormal patterns of transcription with the wild type gene products playing a role in compacting chromatin.

We have demonstrated that human invadolysin localizes to lipid droplets (11). Two recent mass spectrometric analyses of lipid droplets have complemented this observation, with the identification of core histones in the lipid droplet proteome—histones H2A, H2Av and H2B in *Drosophila* embryo lipid droplets (48,49), and H2Az, H2Bb and H4 in lipid droplets from human U937 monocytes (50). Indeed the first suggestion of all four core histones (based on size and solubility properties) in lipid droplets released from growing rat fibroblasts was made 30 years ago (51). Microvesicles composed of lipid particles secreted from cultured hamster sebocytes were also shown to be enriched for histone H3 which was proposed to play an anti-microbial role (52). Thus, we hypothesize that an aspect of the histone ubiquitination cycle may occur in association with lipid droplets, with a role for lipid droplet-associated invadolysin in the turnover of ubiquitinated histones.

ubH2B has been found to be a 5’-enriched active transcription mark, and proposed to be necessary for the relaxation of chromatin, enhancing access to transcription machinery (43,53). In some cases, H3K4me3 and ubH2B exhibit ‘trans-tail’ cross-talk (36). We found however that in wild type polytene chromosomes, ubH2B exhibits little overlap in cytological localization with H3K4me3. And while ubH2B has been linked to regions of high transcriptional activity in human cells (54), it should be noted that (in the context of a ChIP experiment), only ubH2B associated with chromatin would have been analysed. The presence or function of ubH2B in the cytoplasm would not have been addressed.

Intriguingly, whole mount immunostaining of *IX-14^1^*/*not^1^* transheterozygous salivary glands revealed that ubH2B accumulated in the cytoplasm rather than in nuclei. We additionally observed the localization of ubH2B on mitotic chromosomes in *invadolysin* and *nonstop* mutant and *IX-14^1^*/*not^1^* transheterozygous larval neuroblasts. Therefore, we hypothesize that regulating the balance and location of ubH2B are crucial to achieving precise higher order chromosomal architecture.

The accumulation of ubH2B in the cytoplasm of homozygous *invadolysin, nonstop*, and transheterozygous *invadolysin*/*nonstop* cells suggests that the appropriate processing of this ubiquitin modification may play a role in the orderly deposition of histones into chromatin. As newly synthesized histones are characterized by specific acetylation and methylation post-translational modifications, it will be interesting to examine whether these modifications are altered in newly synthesized histones from *invadolysin* or *nonstop* mutant cells (55,56).

### A model for invadolysin's role in histone modification

This is the first time that a genetic interactor of invadolysin (*nonstop*) has been identified and described. In this study, we have examined histone modification by immunoblotting the entire cellular histone complement, and not that of the DNA-associated histones as would be assessed in a ChIP experiment, or acid extracted histones from isolated larval nuclei. In our experiments, we observed an accumulation of both ubH2B and H3K9/14ac in *invadolysin* and *nonstop* mutants and transheterozygotes, and thus postulate that invadolysin may have *opposing* effects on the DUB (deubiquitinating) and HAT (acetylating) modules of the SAGA complex. The molecular reasons for this can only be speculated upon at present, but may have to do with processing or assembly of SAGA subunits. It has been reported that H3K9 acetylation levels were unchanged in acid extracted histones from *nonstop* or *sgf11* mutant nuclei, and decreased in *ada2b* mutant nuclei (27). In this study, H3K9/14ac appeared unchanged in *sgf11*, mildly increased in *ada2b*, and *gcn5* whole larval extracts, but substantially increased in *nonstop* extracts. It is certainly plausible that the complement of histone modifications observed will differ depending on mutant allele analysed or technology utilized.

**Figure 8.**
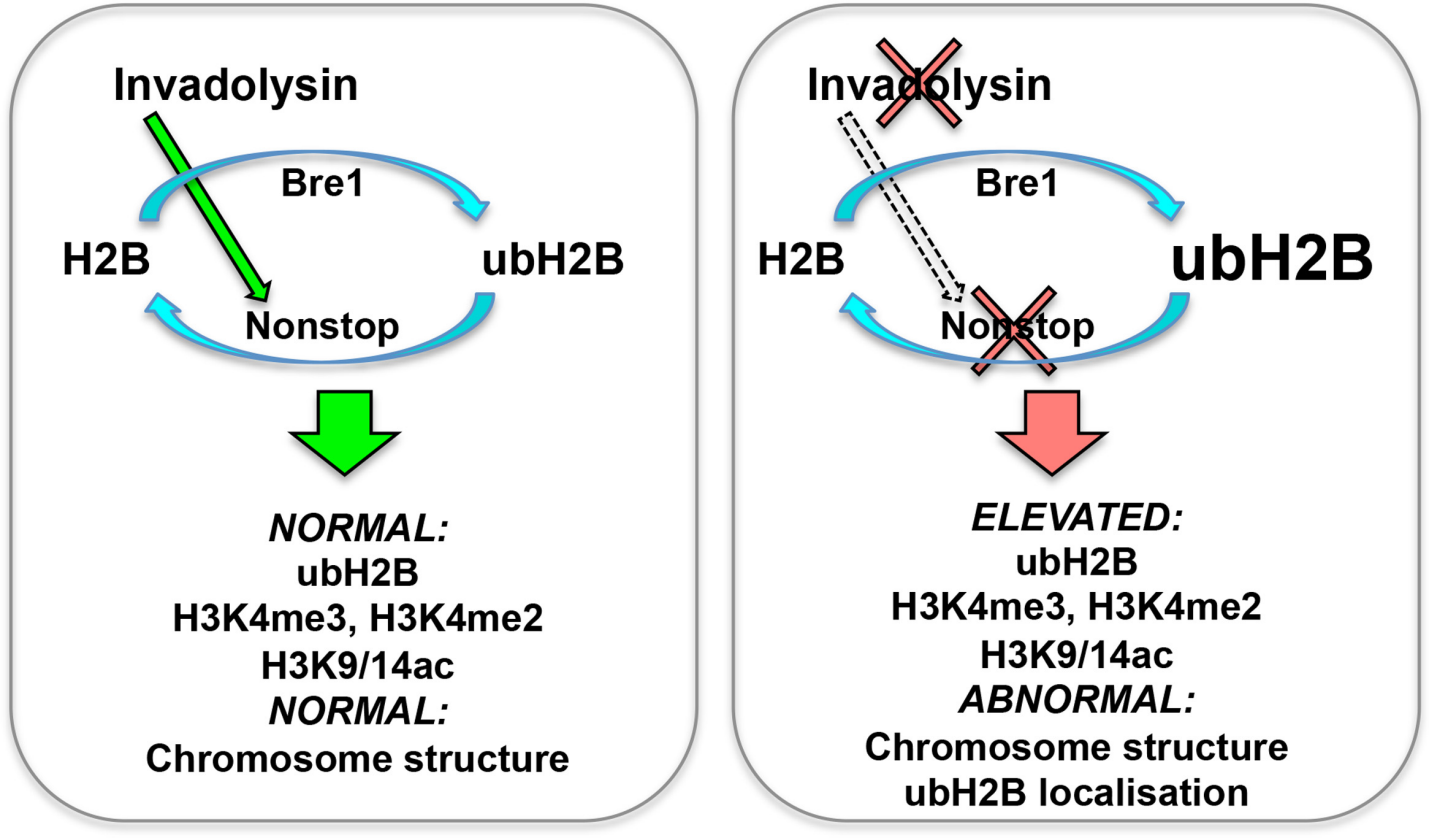
Model for the interaction between *invadolysin, nonstop* and *bre1*. We hypothesize that invadolysin affects higher order chromosome architecture through an effect on the balance of histone modification. Ubiquitination of histone H2B is accomplished through the concerted action of the *bre1* ubiquitin ligase and the *nonstop* ubiquitinating protease. In the absence of invadolysin, we speculate there is decreased activity of nonstop, which would result in the accumulation of ubiquitinated H2B. In *nonstop* and *invadolysin* mutants, and in animals where Bre1 is overexpressed, ubH2B, H3K4me3, H3K4me2 and H3K9/14ac accumulate and abnormally-structured chromosomes are observed.

Based on our results, we propose the model in Figure 8 for the role of invadolysin in higher-order chromosome structure. Invadolysin may work to balance the dBre1 [ubiquitinating] and nonstop [deubiquitinating] activities to regulate histone ubiquitination and ensuing chromosomal architecture. When ubH2B accumulates in the cytoplasm or on mitotic chromosomes, chromosome structure is affected, such that chromosomes appear hypercondensed in length, yet fuzzy in periphery. Cytoplasmic ubH2B accumulation may also result in changes in transcription via the accumulation of H3K4me3. We have not yet determined whether the genetic interaction described herein is also mirrored by direct physical interaction between invadolysin and SAGA complex subunits.

Further studies involving the identification of physical partners of invadolysin, nonstop and ubH2B will be instrumental in understanding how the cycle of H2B ubiquitination ties in ultimately with chromosome structure. Investigation of the presence and detailed dynamics of ubH2B in the cytoplasm will also illuminate the role and/or consequence of mono-ubiquitinated histone H2B in this unexpected location.

## SUPPLEMENTARY DATA

Supplementary Data are available at NAR Online.

SUPPLEMENTARY DATA
