# Supplementary material for: Invadolysin acts genetically via the SAGA complex to modulate chromosome structure
Source: Nucleic Acids Res. 2015 Mar 16;43(7):3546–62. doi: 10.1093/nar/gkv211 (PMC4402531; doi:10.1093/nar/gkv211)
Supplement: SUPPLEMENTARY DATA [file supp_43_7_3546__index.html]

Invadolysin acts genetically via the SAGA complex to modulate chromosome structure — Invadolysin acts genetically via the SAGA complex to modulate chromosome structure — SUPPLEMENTARY DATA 

# Invadolysin acts genetically via the SAGA complex to modulate chromosome structure

## SUPPLEMENTARY DATA

**Files in this Data Supplement:**

- SUPPLEMENTARY DATA
- SUPPLEMENTARY DATA
- SUPPLEMENTARY DATA
- SUPPLEMENTARY DATA
- SUPPLEMENTARY DATA
- SUPPLEMENTARY DATA
