# Supplementary material for: Invadolysin acts genetically via the SAGA complex to modulate chromosome structure
Source: Nucleic Acids Res. 2015 Mar 16;43(7):3546–62. doi: 10.1093/nar/gkv211 (PMC4402531; doi:10.1093/nar/gkv211)
Supplement: SUPPLEMENTARY DATA [file supp_gkv211_nar-03372-x-2014-File010.docx]

**Supplemental Figure 1.**

We utilised Image J to quantitate the immunoblots shown in Figure 2. While the quantitation shown herein is only for the immunoblots shown in Figure 2, the qualitative results have been obtained numerous times (>5). The signal for each antigen was first compared relative to the loading control (α-tubulin), and then normalized relative to the wild type genotype in that immunoblot using Graph Pad Prism version 6. The y-axis in each graph represents the fold change in intensity. The asterisks in the [3^rd^] H3K4me3 panel only serve to show that accurate determination of the fold increase in the *IX-14/not* transheterozygote and upon Bre1 overexpression was not possible as the signal for the wild type was too low to detect (Figure 2B and 2C).

**Supplemental Figure 2.**

Mapping of ubH2B (red) vs H3K4me3 (green) and DNA (blue) on polytene chromosomes. Segments 1/2/3 highlighted in the polytene squash in A are magnified in B. The chromocentre (*) is shown magnified in Figure 5B. Segment 3 is also shown in Figure 5C.

**Supplemental Figure 3.**

Mapping of ubH2B (red) vs H3K4me3 (green) and DNA (blue) on polytene chromosomes. The individual grey-scale images are shown for segments 1/2/3 highlighted in the polytene squash in Supplemental Figure 2.

**Supplemental Figure 4.**

ImageJ pixel analysis of the chromocentre and the three independent regions of polytene chromosome depicted in Supplemental Figure 1 and revealed a distinct negative correlation between ubH2B and H3K4me3, localisation along wild type polytene chromosomes.

**Supplemental Figure 5.**

Immunostaining of brain squashes from *invadolysin*, *nonstop* and *IX-14^1^/not^1^* transheteroygous mutant larvae exhibit an increase in ubH2B staining compared to wild type. In addition, mitotic chromosomes (particularly from *nonstop* neuroblasts) are observed to stain intensely for ubH2B (white arrow).

**Supplemental Table 1. Dm SAGA mutants suppress the ‘rough eye’ phenotype due to *invadolysin* overexpression.**

The resultant phenotype was consistent over 4 crosses with over 80 flies of each genotype observed. Su = suppressor, HAT = histone acetyltransferase, SAGA = Spt-Ada-Gcn5-acetyltransferase. n = 20-40 per genotype and cross. The result indicates a consistent phenotype over 3 crosses.

| **Mutant** | **Gene Function** | **Nature of Lesion** | **Result with**  **OE *inv*** |
| --- | --- | --- | --- |
| ***not^1^*** | *nonstop*; ubiquitin protease, part of the deubiquitination module of dSAGA | EMS-induced mutation, hypomorph [[22](#_ENREF_22)] | **Su** |
| ***not^2^*** | *nonstop*; ubiquitin protease, part of the deubiquitination module of dSAGA | P element insertion (P{PZ]) within coding sequence, null mutant [[55](#_ENREF_55)] | **Su** |
| ***sgf11^e01308^*** | *sgf11*; part of the deubiquitination module of dSAGA, required for proper functioning of nonstop | PiggyBac insertion (PBac[RB]) in the promoter region of *sgf11* [[27](#_ENREF_27)] | **Su** |
| ***ada2B^1^*** | *ada2B*; component of dSAGA involved in positive regulation of HAT activity | 1,077-bp deletion that includes the transcription start site, entire first exon and translation start codon [[56](#_ENREF_56)] | **Su** |
| ***Pcaf^E333st^*** | *gcn5*; catalytic component of dSAGA’s HAT activity | EMS-induced mutation that results in the formation of a stop codon. Mutation found within the first exon [[57](#_ENREF_57)] | **Su** |
