# Supplementary figures and images for: Invadolysin acts genetically via the SAGA complex to modulate chromosome structure
Source: Nucleic Acids Res. 2015 Mar 16;43(7):3546–62. doi: 10.1093/nar/gkv211 (PMC4402531; doi:10.1093/nar/gkv211)

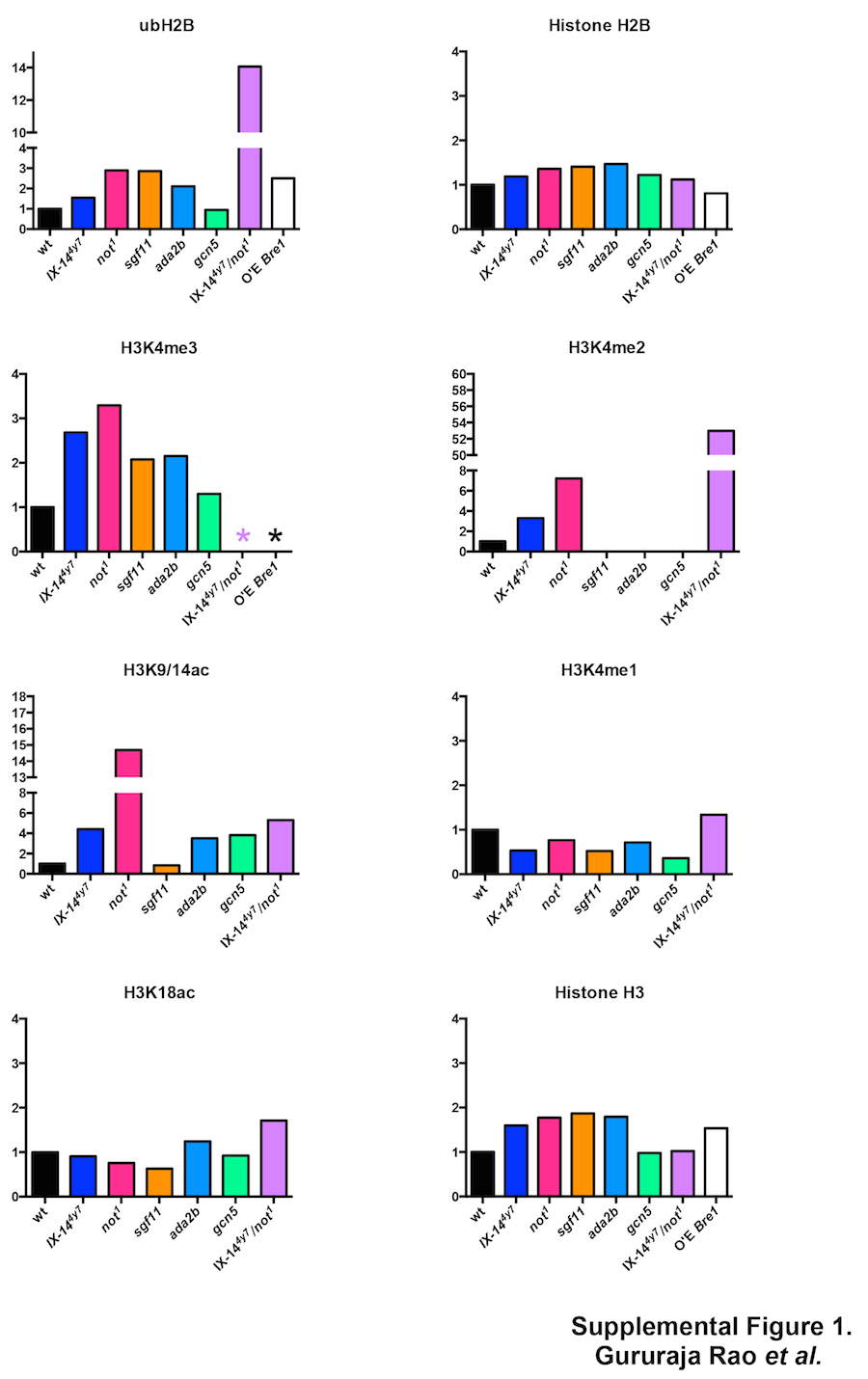

Supplement: SUPPLEMENTARY DATA [file supp_gkv211_nar-03372-x-2014-File011.tif]

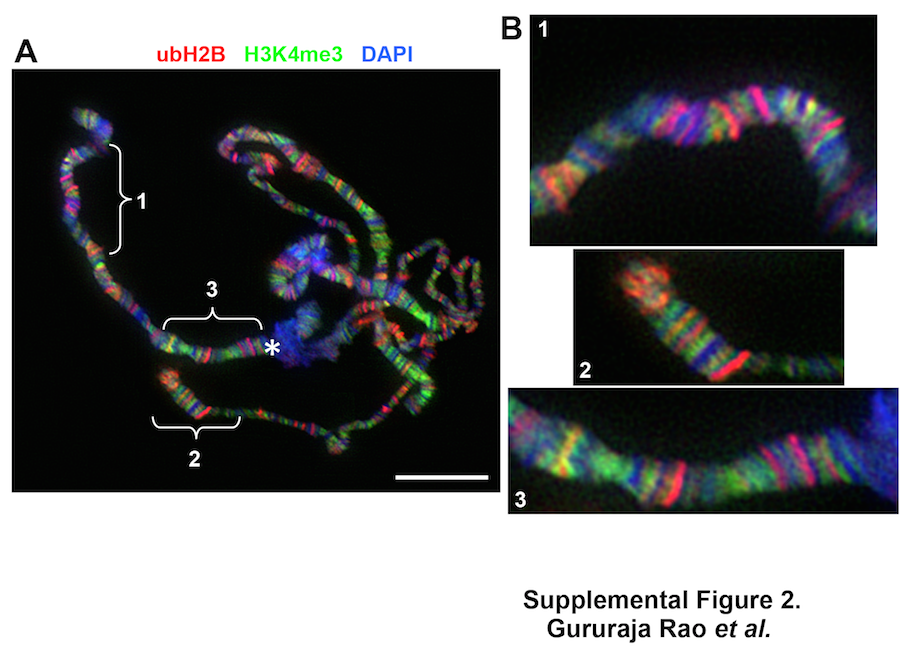

Supplement: SUPPLEMENTARY DATA [file supp_gkv211_nar-03372-x-2014-File012.tif]

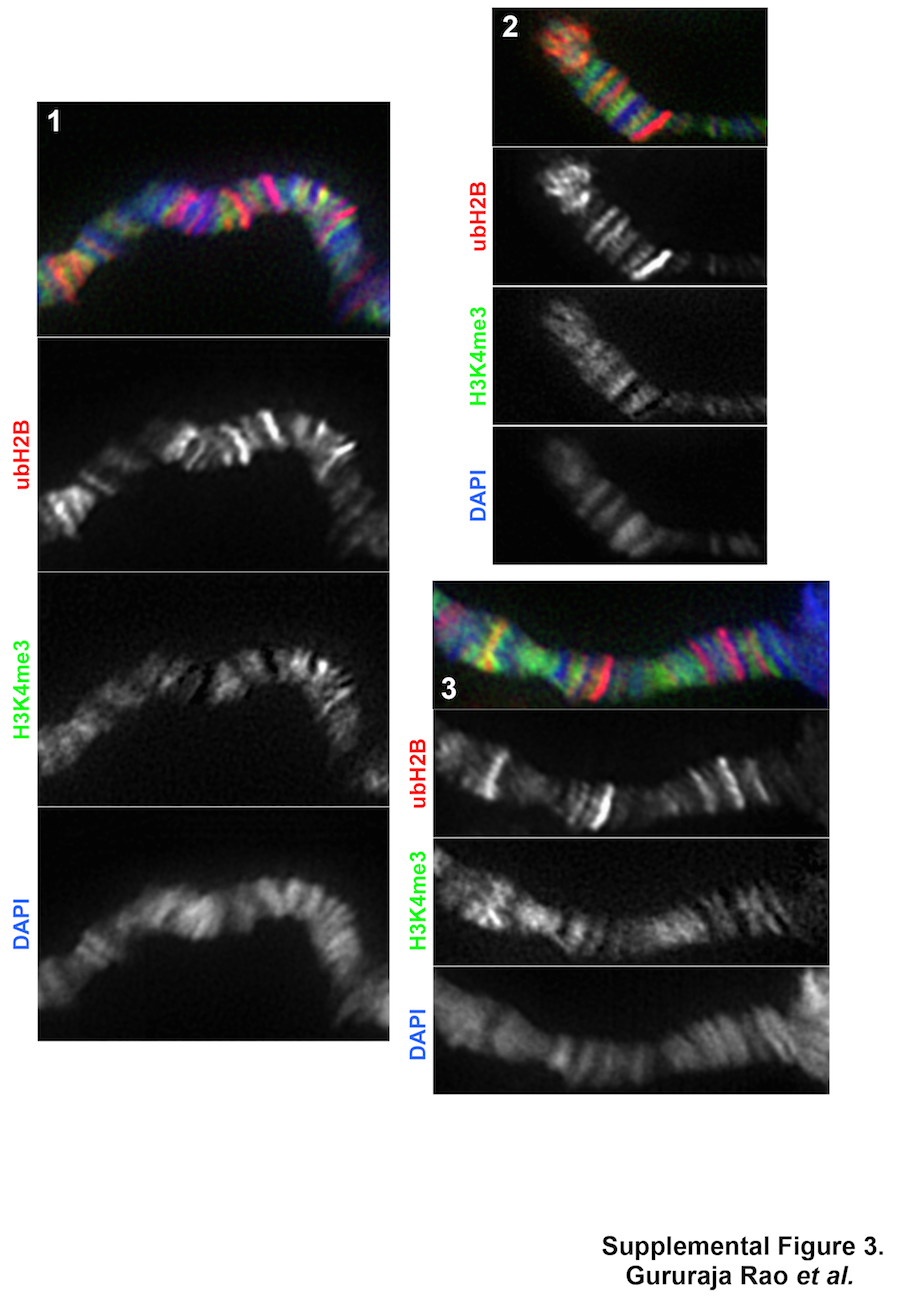

Supplement: SUPPLEMENTARY DATA [file supp_gkv211_nar-03372-x-2014-File013.tif]

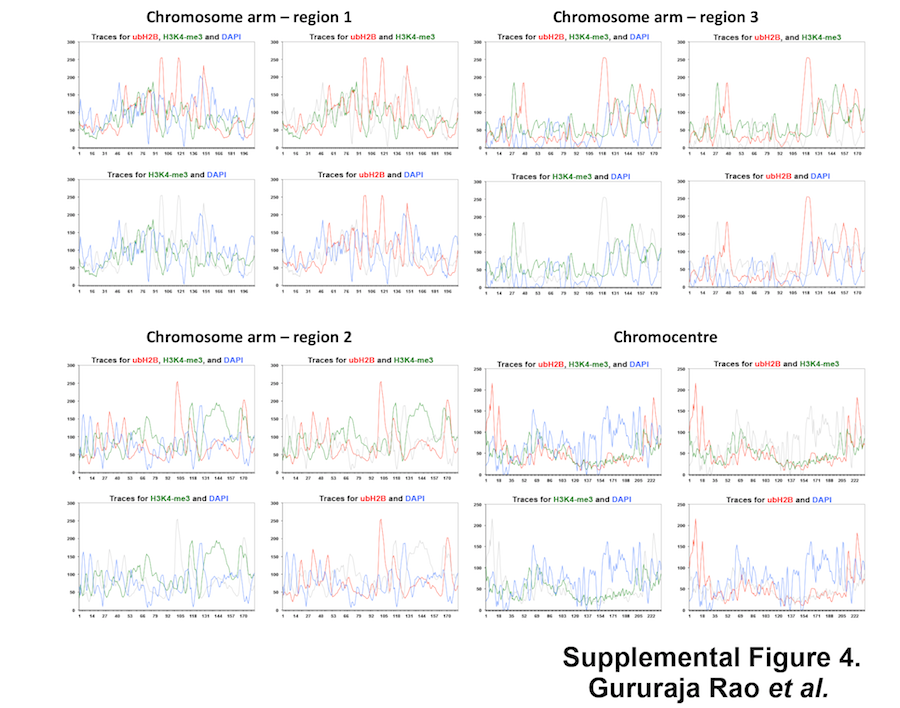

Supplement: SUPPLEMENTARY DATA [file supp_gkv211_nar-03372-x-2014-File014.tif]

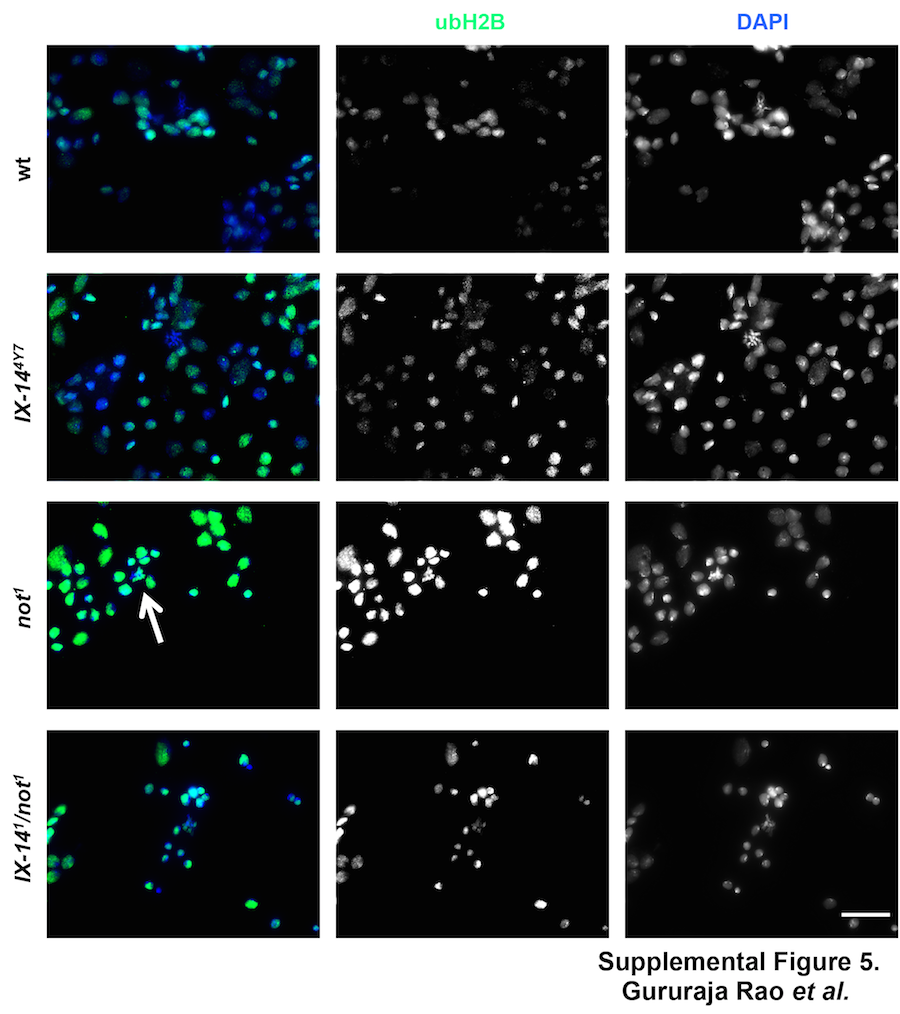

Supplement: SUPPLEMENTARY DATA [file supp_gkv211_nar-03372-x-2014-File015.tif]
